# Clinical and radiographic prospective study of customized one-piece titanium and one-piece fusion-sputtered zirconia implants: five-year mean follow-up

**DOI:** 10.1186/s12903-022-02600-9

**Published:** 2022-11-24

**Authors:** Mahy Hassouna, Walid Al-Zordk, Moustafa Aboshilib, Mohamed Ghazy

**Affiliations:** 1 Fixed Prosthodontics Department, Faculty of Dentistry, Mansoura University, Mansoura, Egypt; 2 Biomaterials Department, Faculty of Dentistry, Alexandria University, Alexandria, Egypt; 3 Faculty of Dentistry, Horus University, New Damietta, Egypt

**Keywords:** Fusion-sputtered, Implant, One-piece, Titanium, Zirconia

## Abstract

**Background:**

To evaluate the clinical and radiographic assessment of customized fusion-sputtered one-piece zirconia implants.

**Methods:**

Twenty-eight patients received either fusion sputtered one-piece zirconia implants (n = 14) or one-piece titanium implants (n = 14). All implants were one-piece designs. After 4 months of immediate loading, all implants were restored with a monolithic zirconia crown. All implants were evaluated at baseline, 6 months, 1 year, 2 years, and 5 years. Implant mobility, plaque index, and gingival index evaluations were performed. The measurements of marginal bone level were calculated radiographically.

**Results:**

All implants were well maintained through the evaluation period with a 100% survival rate without any clinical complications. Regarding gingival index, there was no statistically significant difference (*P* = .364) between zirconia (3.3 ± 0.7 mm) and titanium (3.5 ± 0.6 mm) implants, after 5 years. There was no statistically significant difference (*P* = .470) between zirconia (1.77 ± 0.039 mm) and titanium (1.80 ± 0.28 mm) implants regarding marginal bone loss, after 5 years.

**Conclusions:**

One-piece fusion-sputtered zirconia implant represents a reliable treatment modality in replacing a missing tooth in the esthetic zone.

**Supplementary Information:**

The online version contains supplementary material available at 10.1186/s12903-022-02600-9.

## Background

Every day, more difficult cases are being treated with dental implants, and the success rate is increasing [1]. Since the discovery of osseointegration by Per-Ingvar Branemark in the 1970s, a stress-free healing period of 3–6 months was originally considered a prerequisite for achieving osseointegration of titanium implants [2]. Immediate or early loading protocols have several advantages for patients, including a reduction in the number of surgeries and treatment duration, as well as an increase in patient satisfaction. It also reduces the time between extraction and prosthetic rehabilitation, which may lead to greater patient satisfaction and acceptance. Preservation of a natural soft tissue profile in accordance with interim restoration and enhanced bone maturation are two biological advantages of these procedures [3].

A number of clinical techniques have been introduced in order to reduce the risk of immediate loading, like accurate patient selection, less preparation of the osteotomy, the use of nonfunctional provisional crowns, and the use of implants with special anatomical designs [4]. Increasing the implant load distribution area by using special implant designs for immediate loading and unloading improves primary stability on the day of surgery [5]. The concept of a one-piece implant, which includes both the bone anchoring part and the abutment in a single piece, is not new. Andre Schroeder created and debuted this design in the early 1980s [6].

Excellent biocompatibility and a wide range of therapeutic possibilities are provided by titanium implants [1]. Failure of titanium oral implants may be caused by significant titanium leakage, according to studies on animals. These particles have been observed in nearby lymph nodes and in macrophages linked to failed implants [7, 8]. However, concerns about titanium sensitivity have started to surface recently [9]. The grey tint of titanium is another flaw. The black shadow of titanium may be apparent through the peri-implant tissues when placed in aesthetic locations with a thin gingival biotype, which compromises the aesthetic result [10]. Consequently, the zirconia implant has been introduced as an alternative to the titanium implant. Zirconia exhibits promising properties such as good biocompatibility, low affinity to bacterial plaque, low thermal conductivity, high flexural strength (900–1200 MPa), corrosion resistance, and its non-metallic color [11]. Zirconia was first utilized in dentistry in the form of 3Y-TZP which that contained 0.25–0.5 wt% alumina. By lowering the alumina content, translucent zirconia was created, and the translucency was improved [12–14]. In an effort to improve the surface properties of zirconia, airborne-particle abrasion, acid etching, plasma spraying, aggregation of bioactive materials, UV radiation, and fusion sputtering techniques have been employed to enhance bone apposition and implant stability [15, 16].

Fusion-sputtering is a new technology for creating a rough zirconia surface by spraying small zirconia particles over unsintered zirconia with an air–water jet. These particles get structurally bonded with the zirconia surface during sintering, resulting in a layer thickness of 4 to 12 [17]. In a prior animal investigation, [18] fusion-sputtered zirconia implants had significantly greater mean removal torque values (78.70 ± 2.88 Ncm) than control zirconia (63.64 ± 3.02 Ncm) and when compared to titanium implants (74.96 ± 3.72 Ncm), but the differences were not statistically significant.

After 4 weeks, the existence of newly produced bone trabeculae in direct contact with all implant surfaces could be seen histologically. There were no gaps, fibrous tissue, or foreign body reaction at the implant-bone interface, indicating active osteoblasts secreting osteoid matrix. After 8 weeks of recovery, there was a further rise in bone apposition on all implant surfaces. Successful osseointegration of zirconia and titanium implants was found across the whole length of all implant surfaces after 12 weeks. At the examined intervals, histomorphometric analysis revealed no significant difference in measured bone density within or outside the implant threads between fusion-sputtered zirconia and titanium implants [18].

Since immediate loading of one-piece implants has become a widely used procedure for the rehabilitation of partially edentulous patients, no reports are available on the impact of fusion sputtering implant surface treatment on the clinical outcome of zirconia implants. Therefore, the aim of this clinical investigation was to evaluate and to compare the safety and efficiency of fusion-sputtered customized one-piece zirconia dental implants in comparison with titanium implants after 5 years of loading. The proposed hypothesis tested in this study was that fusion sputtered zirconia implants would perform similarly compared to one-piece titanium implants.

## Materials and methods

### Study design and patient selection

The study was prospective cohort study, comparing one-piece zirconia implants and one-piece titanium implants, for the replacement of a single-tooth in the maxillary premolar area. Sample size calculation was based on success rate between cases with zirconia and titanium implants retrieved from previous research. Using G*power version 3.0.10 with expected difference of 49%, 2-tailed test, α = 0.05 and 80.0% power. The total sample size was 14 cases at least in each group (28 cases total). For randomization, the patients were sorted by using online software (www.sealedenvelope.com). A blocked list was generated and a randomization code was performed. A staff member not involved in the study prepared the envelopes.

Between the years 2016 and 2017, a total of 28 healthy patients in need of implant-supported single tooth restorations in the maxillary premolar area were included in this according to certain inclusion and exclusion criteria.

Informed consent was obtained from all patients prior to the start of the study. The study was conducted at Faculty of Dentistry, Mansoura University, Egypt after the approval of the ethics committee. All patients were asked to participate in the investigation in consecutive order, provided they fulfill the following inclusion criteria: systemically healthy and willing to comply with the planned regimen, in need of implant-supported single tooth restoration for missing maxillary premolar, have sufficient bone height, width, and density to receive implants, has sufficient soft-tissue volume and the opposing dentition is natural teeth. The exclusion criteria included the followings: alcohol or drug abuse, smoking of more than 10 cigarettes per day, health conditions that do not permit the surgical procedure, medical conditions requiring prolonged use of steroids, medications that could interfere with bone metabolism, metabolic bone disorders, history of neoplastic disease requiring the use of radiation or chemotherapy, physical handicaps that would interfere with the ability to perform adequate oral hygiene, the subject is not able to give her informed consent to participate, untreated or active periodontitis, mucosal diseases, unhealed extraction sites, the need of bone augmentation, bruxism or other destructive habits.

### Fabrication of zirconia implants

**Fig. 1:**
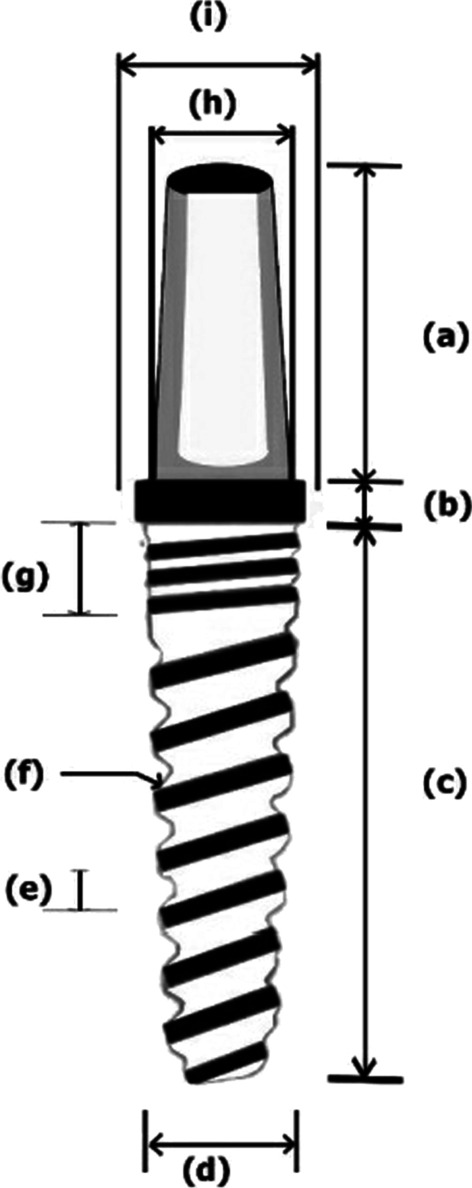
Diagram representing implant design. **a** Abutment portion height (6 mm), **b** trans-mucosal portion (1 mm), **c** implant body length (12 mm), **d** domed apex diameter (2.6 mm), **e** thread pitch (1 mm), **f** thread width (0.5 mm), **g** microthreads in the cervical portion of the implant body, **h** abutment diameter (3.4 mm), and **i** trans-mucosal portion diameter (3.9 mm)

**Fig. 2:**
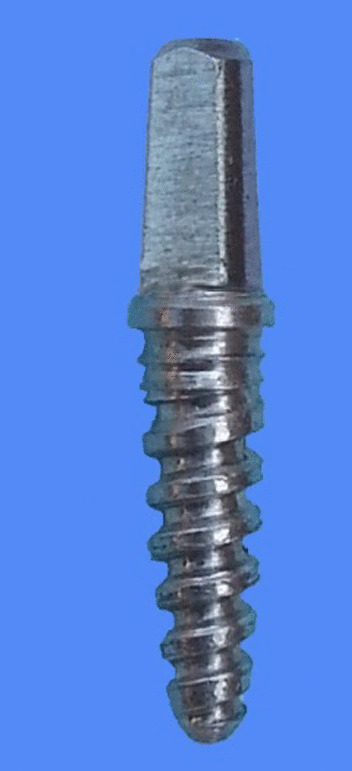
Stainless steel model

The present study investigated a newly developed customized zirconia dental implant with the novel fusion-sputtered surface treatment. All the implants were manufactured with the same design, diameter and length. The manufacturing process was started with the fabrication of a stainless-steel model (Figs. 1 and 2) which had a diameter of 3.7 mm tapering toward the apex to be 2.6 mm with a length of 12 mm, and the abutment portion had a diameter of 3.4 mm and length of 6 mm with anti-rotational aspect. The fixture had spiral threads with 1 mm pitch and 0.5 mm width. The stainless-steel model was coated with the scanning spray (Arti-Scan CAD/CAM Spry, Bouch GmbH Co., Germany) in order to achieve a scanning surface. The model was scanned with the optical scanner (Ceramill Map 400, Amann Girrbach GmbH, Germany) following the software instructions. Based on the scanning data, the zirconia implants were designed using CAD/CAM software (Ceramill Mind CAD version 3.5.6.1408, Amann Girrbach GmbH, Germany). Based on the scanning data, the implants were milled from Ceramill zolid preshades blocks (HT Zirconia) (Ceramill ZI; AmannGirrbach GmbH, Germany) using a 5-axis milling machine (Ceramill Motion 2–5 axis, Amann Girrbach GmbH, Germany). These blocks are made of tetragonal polycrystalline zirconium oxide stabilized with 2–3 mol% yttrium oxide. After the milling process, each implant was separated from the block and the point of attachment was smoothened.

### Fusion sputtering

**Fig. 3:**
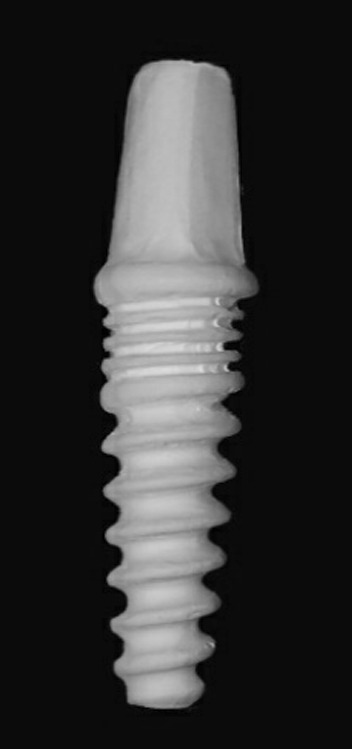
Customized one-piece zirconia implant before sintering

**Fig. 4:**
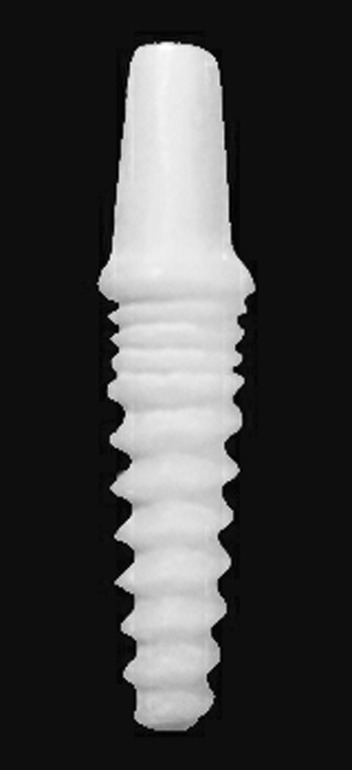
Customize one-piece zirconia implant after sintering

**Fig. 5:**
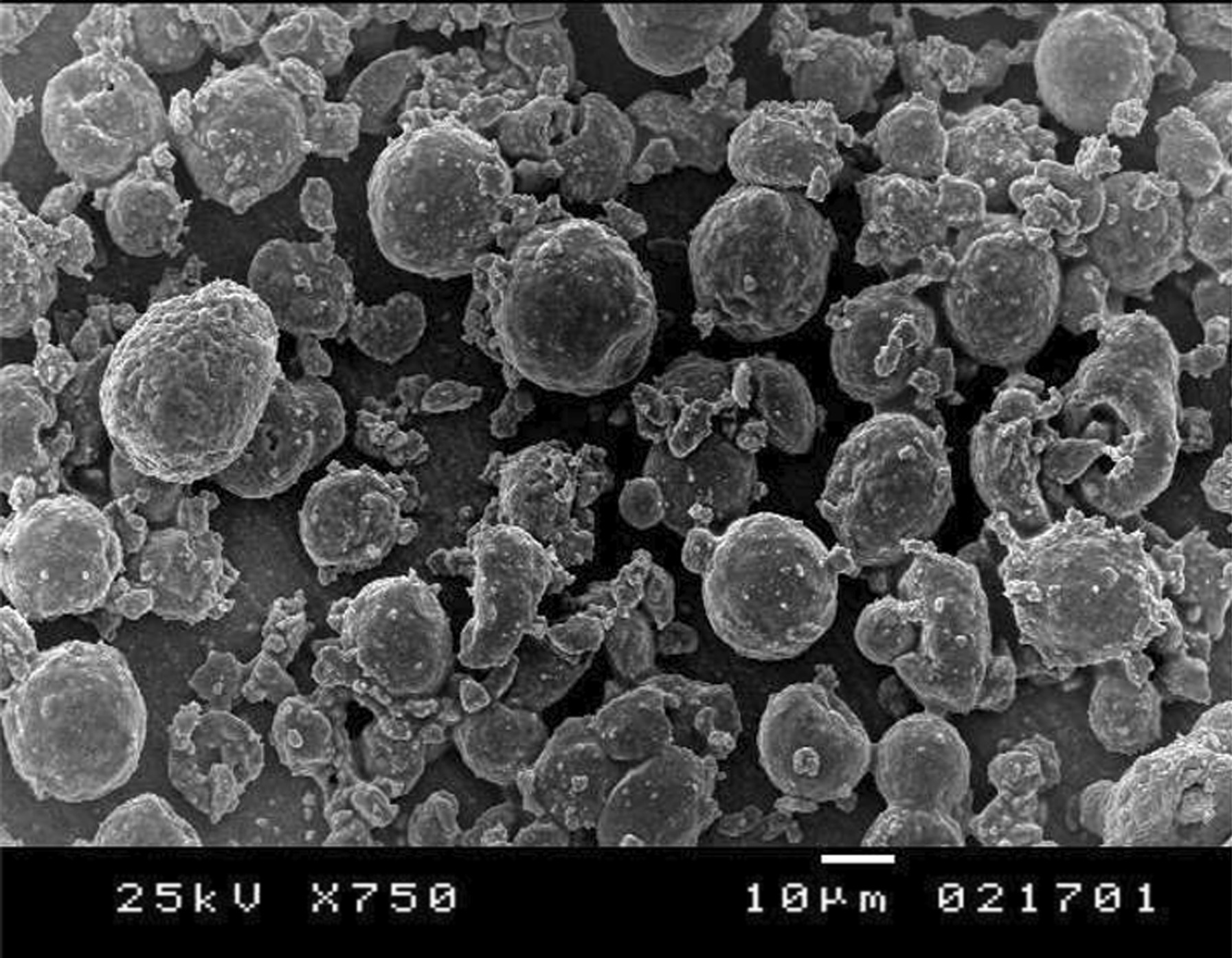
Scanning electron microscopic image of fusion sputtered zirconia implants demonstrating creation of beaded surface

All zirconia implants received fusion sputtering [17, 18] surface treatment through spraying a suspension of zirconia mixture composed of 5 gm ultrafine zirconia powder (1–5 µm) and 10 mL ethyl alcohol (70%). To ensure good adherence, 1 mL of polyethyl alcohol was added to the mixture. The slurry was mixed over a stirring plate to produce a homogenous mixture that was sprayed under a pressure of 1 bar on the outer surface of the partially sintered zirconia implants (Fig. 3). Then, they were sintered in a sintering furnace (Ceramill Therm, Amann Girrbach GmbH, Germany) at 1450 °C. After sintering, the surface of zirconia implants (Fig. 4) was examined using a scanning electron microscope (XL30, Philips, Eindhoven, The Netherlands) to ensure proper surface architecture (Fig. 5). The abutment portion was sandblasted.

### Fabrication of titanium implants

Titanium one-piece implants were also customized with the same design, diameter and length. Grade IV titanium rods, 4.5 mm in diameter, were used to mill the required titanium implants as previously described for zirconia implants. All titanium implants were airborne particle abraded using 50 μm alumina particles using 1 bar pressure for 30 s followed by ultrasonic cleaning for 30 min. All titanium implants received acid etching using 2.5% hydrofluoric acid for 5 min followed by 60 min of ultrasonic cleaning in demineralized water applied for 3 cycles. All prepared implants were sterilized using steam autoclaving.

### Surgical interventions

Before implant surgery, the patient received antibiotics (2 × 625 mg Augmentin®, Galaxo Smith Kline Co, Egypt) Patients were instructed to rinse with chlorhexidine (Orovex, Macro-International Co., Egypt). Surgery was done under local anesthesia (Ubisresin forte, 3 M ESPE, United states). The incision was placed at the mid-crest, with releasing incisions if necessary, and a mucoperiosteal flap was raised. Osteotomy preparation started with a pilot hole drill followed by a 2 mm depth drill till the length of 12 mm at 800 rpm. The drilling was done in a straight up and down motion using a low-speed, high-torque handpiece. Subsequently, the osteotomy was enlarged using a 2.5 mm twist drill at 12 mm length followed by a 3.4 mm twist drill till one-third of the implant length.

**Fig. 6:**
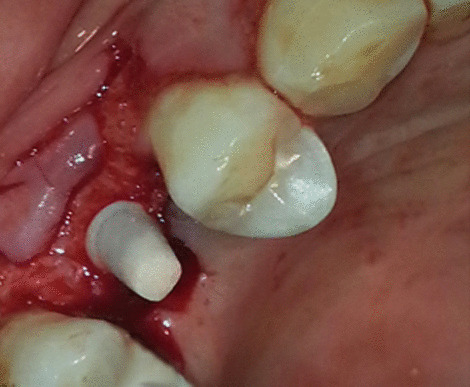
Zirconia implant inserted in surgical site

After osteotomy preparation, the customized one-piece zirconia and titanium one-piece implant was selected (Table 1) and seated in the prepared site using a driver and ratchet wrench (Fig. 6). The flap was repositioned and sutured leaving the abutment of the implant projecting through the mucosa into the oral cavity. The patients were instructed to rinse twice daily with an aqueous solution of 0.2% chlorhexidine and to continue the antibiotic regimen for 5 days. In addition, analgesics (Bi-profenid 150 mg, Sanovi Aventis, Egypt) were prescribed for the next 2 days according to individual needs. Patients were also instructed to refrain from mechanical plaque removal in the area of implantation for 1 week. The sutures were removed 7 to 10 days following implantation.

**Table 1:** Distribution of titanium one-piece and zirconia one-piece implants regarding the number of patients and site of implant placement

	Titanium one-piece implants		Zirconia one-piece implants	
	Number of patients^a^	Site of implant	Number of patients	Site of implant
	4	14	3	14
	3	15	5	15
	3	24	4	24
	4	25	2	25
Total			14	14

48 h after implant placement, the provisional restoration was inserted for each implant with no occlusal contact. Four months later, the definitive restoration was inserted for each implant

^a^FDI tooth-numbering system

### Loading protocol

According to classification of Misch et al. [3], nonfunctional immediate restoration loading protocol was done. After 48 h, the occlusion was evaluated to confirm the presence of sufficient inter-occlusal space. An impression was taking using addition silicon material (Ormaplus Cap. Soc. Euro R.E.A. 829391 C.C.I.A.A. Torino). Within 48 h, a provisional crown restoration (Protemp, 3M ESPE AG, Germany) was fabricated and cemented using eugenol-free provisional luting cement. The excess cement was carefully removed and care was taken to ensure that the margin of the provisional crown did not impinge on or irritate the soft tissue.

### Prosthetic fabrication

Four months later, after soft tissue maturation the provisional was removed. The implant was checked. The abutment portion of the implant was modified and the inter-occlusal space was adjusted if needed. The preparation was done using a high-speed carbide bur (C31A012 Carbide Jota AG, 9464 Ruth, Switzerland) and finished with the high speed tapered diamond (Eterna, Bredent, Senden, Germany). Excessive water cooling was used during abutment modification to protect the implant from overheating. After digital scanning, monolithic zirconia crowns were milled (Ceramill Zolid Preshade HT, Amman Girrbach GmbH, Germany) using a CAD/CAM system (Ceramill motion 2–5 axis, Amman Girrbach GmbH, Germany). After intra-oral checking, the finished restorations were cemented using self-adhesive resin cement (Maxcem Elite, Kerr, Italy).

### Examinations and analyses

The baseline for the radiological analyses was implant placement. Radiographs were taken at implant placement, prosthetic insertion (4 months), 6 months, 1 year, 2 years, and 5 years' follow-up. Periapical radiographs were taken without standardization to evaluate marginal bone level at each interval.

**Fig. 7:**
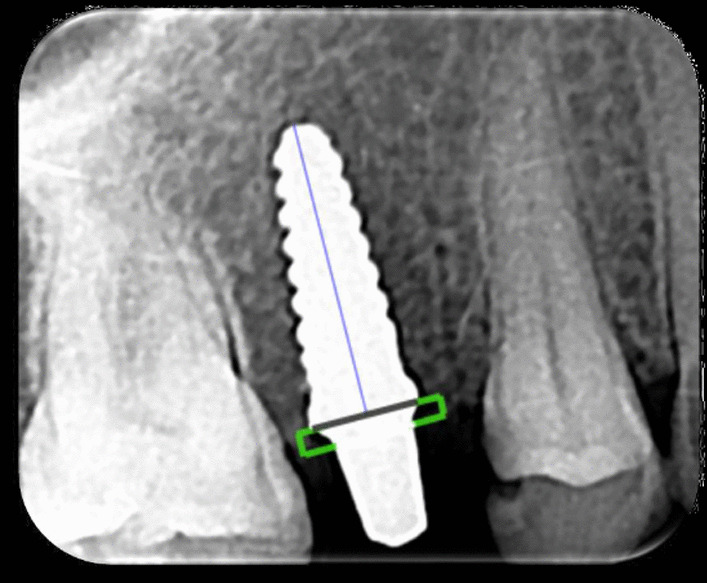
Measuring the marginal bone loss

After radiographic exposure the sensor was scanned with a radiographic dental scanner (Durr-vista scanner, Germany) then the digital radiograph was imported to a scanora software for measurements. The changes of marginal bone the implant was assessed. The average of bone loss on mesial and distal level around side of the implant was measured and recorded in patient chart. To eliminate the radiographic magnification error, the scale of measurement was adjusted according to the actual length of the implant. Two reference points were established within the implant head (one mesial and one distal) at the chamfer finish line of the abutment portion. A straight line was established between the two reference points represented by black line (Fig. 7). Mesial and distal perpendicular lines were created running from this line to the crestal bone. The distance from implant finish line to crestal bone level was measured and analyzed at × 20 magnification using a software program (CorelDraw 10; Corel Corp and Coral Ltd, Ottawa, Canada). This distance represented the marginal bone loss. The known length of the implant (measured from the implant shoulder to the implant apex) according to the manufacturer’s dimensions (12 mm) of the respective implants was used as reference point. To account for variability, the implant dimension (length) was measured and compared to the documentation dimensions; and ratios were calculated to adjust for distortion. Bone levels were determined by applying a distortion coefficient. The actual bone level measurement was performed independently by 2 examiners. The average from both examiner calculations was used as marginal bone level value.

The baseline for the clinical analyses was prosthetic insertion. The clinical evaluation was done at implant placement, prosthetic insertion (4 months), 6 months, 1 year, 2 years, and 5 years' follow-up. Implant stability, absence of pain, perimplant clinical indices (plaque index, gingival index) (Table 2) [19], probing depth (PD), and bleeding on probing (BoP) were evaluated Clinical parameters were recorded mesially, distally, buccally, and lingually at implants. The mean difference of data at baseline (prosthetic insertion) and the respective evaluated time-points was calculated.

**Table 2:** Criteria for the assessment of plaque index and gingival index

Scores	Plaque index^a^	Gingival index^a^
0	No plaque	Normal mucosal aspect
1	A film of plaque adhering to the free gingival margin which can be scrapped of but is not visible	Mild inflammation—slight change in color and slight edema but no bleeding on probing
2	Visible plaque accumulation on the mucosal margin and/or the adjacent tooth surface	Moderate inflammation. Presence of redness, edema, and bleeding on probing
3	Presence of abundant plaque within the gingival sulcus and/or the adjacent tooth surface	Sever inflammation with a marked redness, edema, and bleeding on probing

^a^According to Lang et al. [19]

### Statistical analysis

The data was tabulated, coded then statistically analyzed using IBM SPSS (Statistical package for social science) computer software 2013, version 22.0, Armonk, NY, IBM Corp. Qualitative data were described using numbers and percentages. Quantitative data were described using median (minimum and maximum) for non-parametric data and mean, and standard deviation for parametric data after testing normality using the Shapiro–Wilk test. The significance of the obtained results was judged at the (0.05) level. For qualitative data, the description of the data was done in form of frequency and proportion. Kruskal Wallis and Mann Whitney tests were used to comparing between the two groups. For quantitative data, the description of the data was done in form of means ( ±) SD. General Linear Model measure one-way ANOVA was used to compare one group at different times.

## Results

**Fig. 8:**
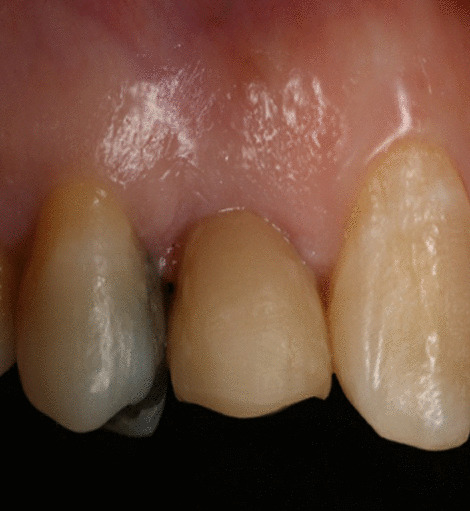
Zirconia implant after 5 years

All implants were well maintained through the period of evaluation with a 100% survival rate with no signs of pain, swelling, pus discharge, or mobility during the observation period (Fig. 8).

Regarding plaque index, zirconia implants resulted in score 0 for 11 implants (78.6%) and score 1 for 3 implants (21.4%), whereas titanium implants scored 0 for 11 implants (78.6%), scored 1 for 2 implants (14.3%) and scored 2 for 1 implant (7.1%). Kruskal Wallis test showed that there was no statistical significant difference as regard to plaque index of one-piece zirconia implant as compared to one-piece titanium implants.

With Gingival index, zirconia implants scored 0 for 11 implants (78.6%) and scored 1 for 3 implants (21.4%), whereas titanium implants scored 0 for 11 implants (78.6%), scored 1 for 2 implants (14.3%) and scored 2 for 1 implant (7.1%). Kruskal Wallis test showed that there was no statistical significant difference as regard to gingival index of one-piece zirconia implant as compared to one-piece titanium implants. Mann Whitney showed that there was no statistical significant difference as regard to plaque index and gingival index at 3 months, 6 months, 12 months and 18 months for one-piece zirconia and titanium implants (*P* = 1.000).

Regarding probing depth after 5 years, there was no statistically significant difference (*P* = 0.364) between zirconia implants (3.3 ± 0.7 mm) and titanium implants (3.5 ± 0.6 mm). The mean probing depth values at follow-up intervals are summarized in Table 3.

**Table 3:** Probing depth (mm) of tested groups

	Zirconia implant	Titanium implant	*P value*
	Mean ± SD	Mean ± SD	
Baseline	2.7 ± 0.6	2.9 ± 0.7	1.000
6 Months	2.9 ± 0.6	3.1 ± 0.7	.218
12 Months	3.2 ± 0.7	3.5 ± 0.6	.330
2 Years	3.6 ± 0.5	3.6 ± 0.5	.395
5 Years	3.3 ± 0.7	3.5 ± 0.6	.364
*P value*	< .001		

**Fig. 9:**
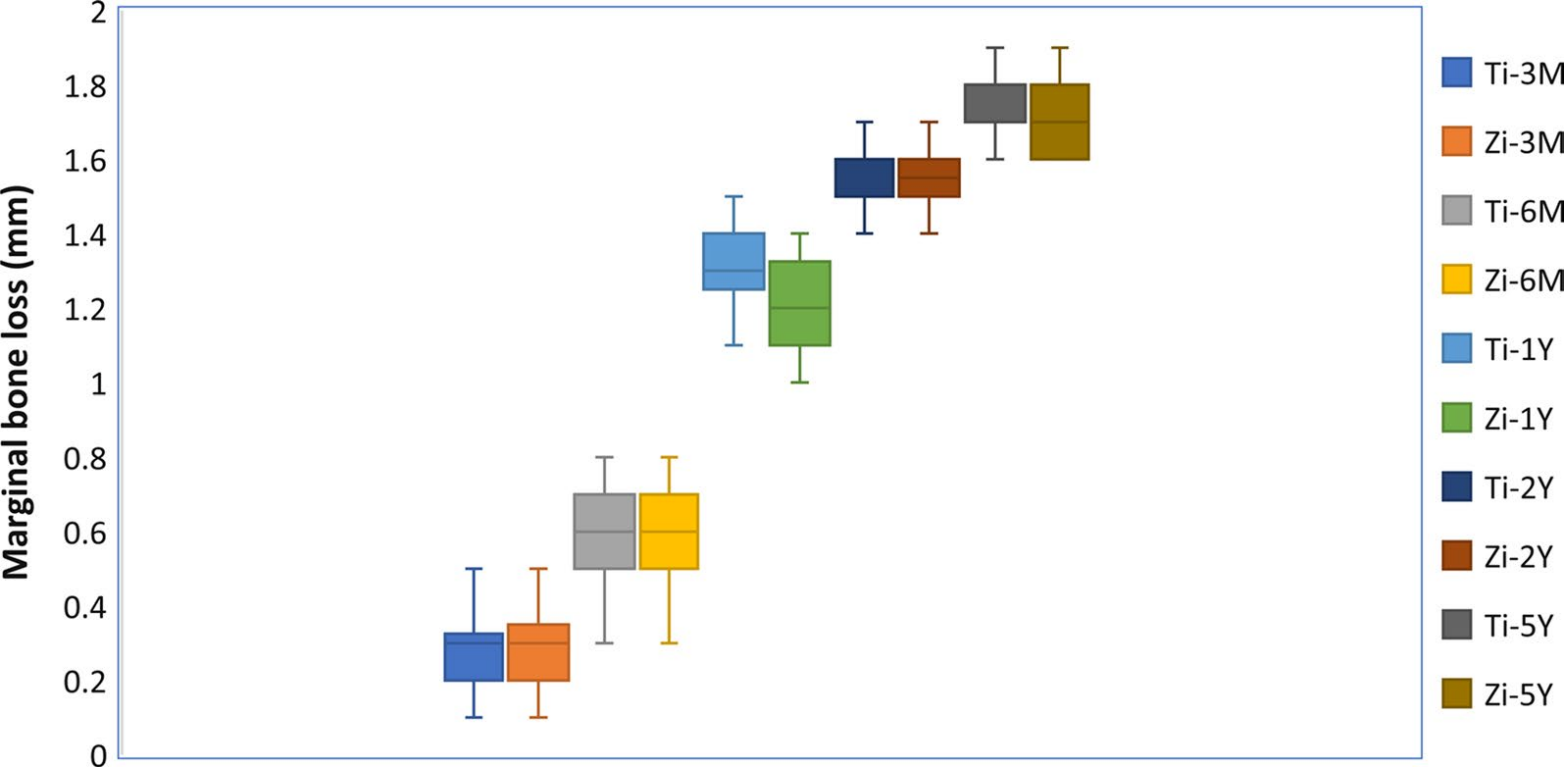
Box plot graph for marginal bone loss for one-piece titanium and one-piece fusion sputtered zirconia implants at 3 months, 6 months, 1 year, 2 years, and 5 years

**Fig. 10:**
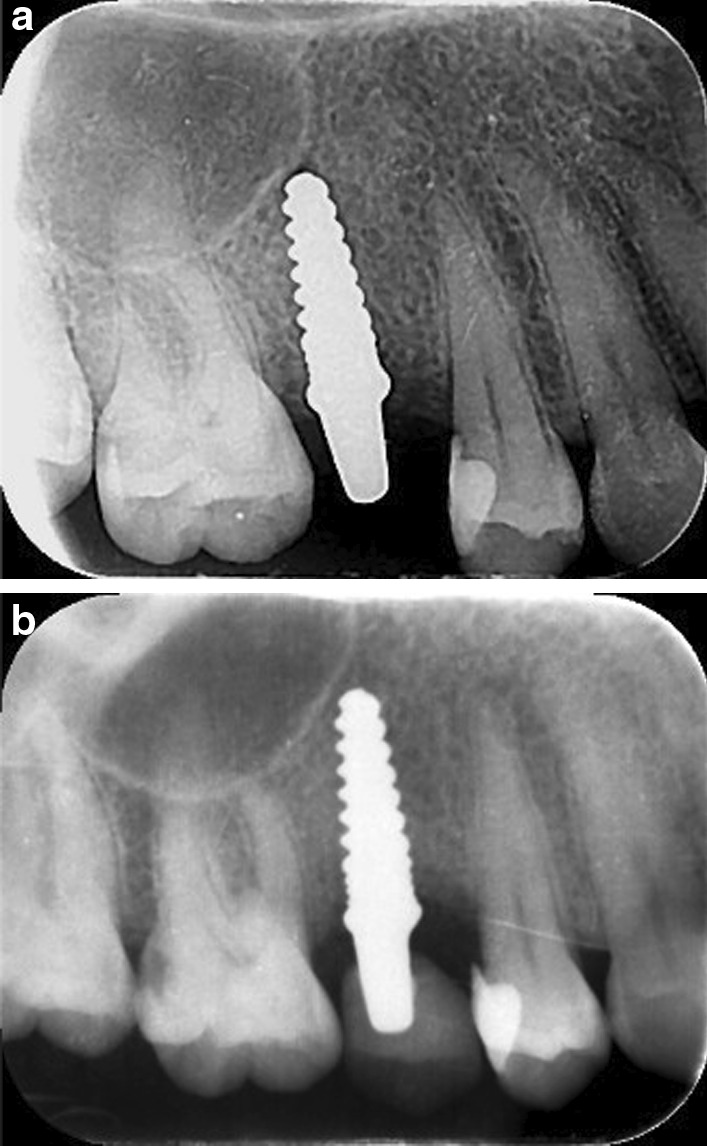
**a** Periapical radiographs of titanium implant after placement. **b** Periapical radiographs of titanium implant after 5 years

**Fig. 11:**
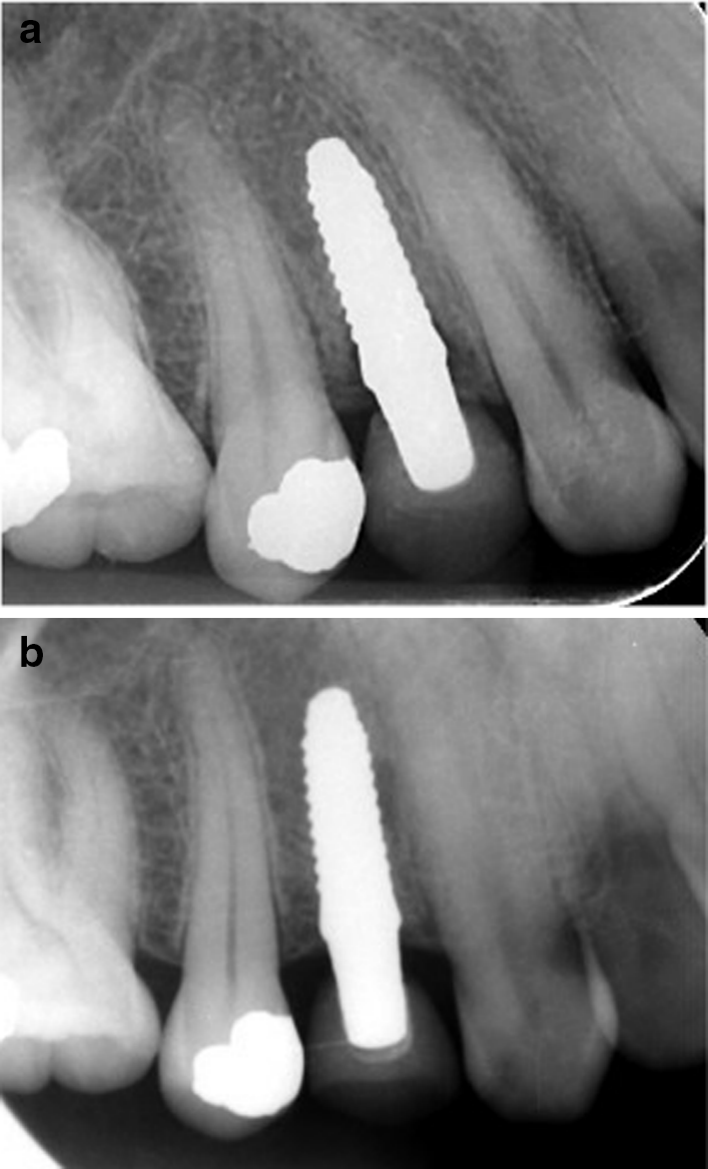
**a** Periapical radiographs of zirconia implant after 1 year. **b** Periapical radiographs of zirconia implant after 5 years

**Fig. 12:**
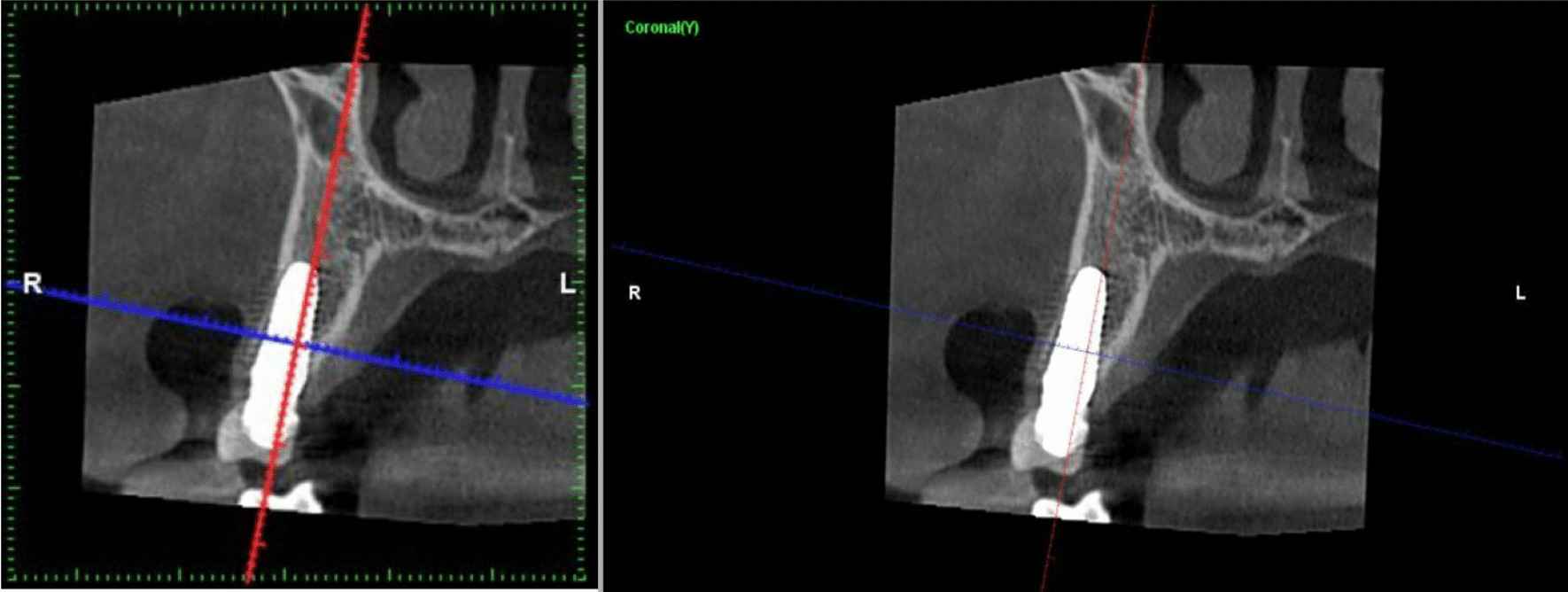
Computed tomography of zirconia implant after 5 years follow up

Marginal bone loss at evaluation periods is represented in Table 4 and Fig. 9. There was no significant difference (*P* = 0.470) between zirconia implants (1.77 ± 0.039 mm) and titanium implants (1.8 ± 0.028 mm), (Figs. 10 and 11). The cone-beam computed tomography was performed after 5 years' follow-up to assess osseointegration of one-piece zirconia implant. (Fig. 12).

**Table 4:** Marginal bone level (mm) at different evaluation intervals

	Zirconia implants		Titanium implants		*p value*
	Mean	Std. Deviation	Mean	Std. Deviation	
3 Months	0.285	0.037	0.285	0.037	1.000
6 Months	0.657	0.073	0.657	0.037	1.000
12 Months	1.300	0.040	1.400	0.040	1.000
2 Years	1.514	0.024	1.603	0.024	1.000
5 Years	1.77	0.039	1.800	0.028	.470
*p value*	˂.048		˂.001

## Discussion

The purpose of this study was to assess the clinical and radiographic outcome of customized fusion-Zirconia has proven to be an excellent titanium substitute. The proposed hypothesis for this study was accepted as there was no significant difference between zirconia and titanium implants regarding probing depth and marginal bone loss. Currently, one-piece designs make up the bulk of zirconia implants [20]. Quality control and correct treatment of the material appear to be of the utmost importance, according to Sadowsky et al. [21]. Zirconia's strength is affected by surface alterations of any kind, including grinding, sandblasting, even little scratches and notches. For osseointegration to occur, there must be an initial engagement between the cells and the implant surface [22]. Studies on animals corroborated the importance of surface roughness on bone apposition and indicated increased efficacy of roughened zirconia implants [15, 23, 24]. Fusion-sputtered zirconia was used to roughen the surface of the zirconia by creating a beaded surface with microscopic porosities, minimizing the surface damage caused by surface modification, and demonstrating a level of osseointegration and interfacial biomechanical stability superior to titanium implants [17, 18].

With immediate loading becoming more and more common, the one-piece implant design gives an intriguing alternative. Immediately loaded zirconia single-piece implants had a good success rate in terms of clinical and radiological outcomes, according to Steyer et al. [25]. The original purpose of this design was to get rid of the structural weakness that the two-piece design had [26]. This design has various benefits, including mechanical strength, no microgaps between the implant and the abutment, fewer surgical operations, and compliance with traditional crown and bridge procedures [26, 27]. These are only a few of the advantages it offers. Customized one-piece implants were created in the current investigation using zirconia and commercially pure titanium. Custom-made implants can now be created thanks to modern technology [28–30].

Customized root-analog zirconia implants have emerged as a potential substitute for standard implants in order to address issues with stress distribution, aesthetics, and biofilm-induced peri-implantitis [31]. With perfect functional and aesthetic results, Figliuzzi [32] demonstrated the successful clinical usage of a custom-made root-form implant. Direct laser metal formation was used to create their titanium alloy implant, which involves the laser-induced fusion of titanium microparticles.

Steam autoclaving was used to sterilize the custom-made implants in this investigation. The surface free energy and surface chemistry of zirconia changed after sterilizing procedures, according to Han et al. [33]. The type of sterilizing process utilized for zirconia has a significant impact on the amount of biofilm that forms on the zirconia surface. They also found that zirconia samples treated with dry heat sterilization had less biofilm formation, but zirconia samples treated with UVC and ray irradiation had more bacterium formation on the zirconia surface than those treated with dry heat sterilization. Both flap and flapless approaches are used in the surgical procedure for implant implantation [33]. The surgical strategy used in this study for implant implantation was flap surgery. The surgical field was better seen with flap elevation, which reduced the risk of bone fenestration and dehiscence. Flap elevation made it easier to analyze bone shape and made it possible to do any necessary alveoloplasty. The flapless approach offers a number of possible drawbacks. It's more difficult to see anatomical landmarks and vital structures with flapless, there's a higher risk of thermal trauma to the bone due to limited external irrigation during osteotomy preparation, and there's a chance of implant surface contamination or deposition of epithelial or connective tissue cells in the osteotomy, which can hamper osseointegration [34].

The abutment part was adjusted in situ in this investigation. After heating bone to 47–50 °C for one minute, tissue injury can occur [35, 36]. Excessive bone resorption has been found in previous research about one-piece implants, and several reasons have been proposed to explain this, including the required in situ preparation [37, 38]. After in situ preparation, Fine et al. [39] observed steady marginal bone level and healthy soft tissue, showing that the one-piece implant can sustain hard and soft tissue health.

The amount of heat created during intra-oral abutment preparation under coolant did not cause bone loss or promote osteoclastogenesis at the bone-implant contact, according to Russe et al. [40]. The participants in this study were given a provisional crown without occlusal contacts, which was replaced by the final restoration 4 months later. The provisional crown was designed free from direct occlusal contact with opposing dentition. The benefit of the transient initial period is adaptation to the improved function and soft tissue stabilization. Previous research has found no significant difference in implant longevity or bone resorption between immediate implant loading with and without occlusal contact [41].

For the purpose of cementation of each crown to its corresponding abutment on the one-piece dental implant, self-adhesive resin cement was used. In contrast to traditional glass-ionomer cement and resin-ionomer cements, it has high compressive strength, low solubility, and resists tensile fatigue. This kind of cement eliminates the need for bonding agents, which speeds up the bonding process and, most critically, reduces the "window of contamination." Due to the presence of phosphate ester group in its makeup, it chemically linked to etched ceramic and zirconia ceramic. According to Rohr et al. [42], self-adhesive and adhesive composite resin cements nevertheless show great crown retention even though they have only a weak bonding ability to zirconia.

Rutkowski et al. [43] reported that zirconium dioxide implants were able to accomplish comparable survival and success rates and patient satisfaction to titanium implants even if they were immediately or delayed loaded. The plaque accumulation and bleeding on probing were within the range of previous studies [44]. In this clinical study, the soft tissue response to zirconia implants was better than titanium implants, which is in agreement with Scarano et al. [45] who showed that a significant reduction in the number of bacteria was observed for the surface of zirconia compared to titanium and Balmer et al. [46] who concluded that one-piece zirconia implants are considered reliable for the construction of implant-supported restoration with a stable mucosal margin level after 5 years of follow up. A positive correlation has been found between oral hygiene and bone resorption around implants [47]. The reduction of bacterial adhesion on the zirconia surface enhances the formation of a mucosal seal that inhibits early marginal bone loss.

This study verifies the predicted outcome of immediate loading with a one-piece implant design, with an implant survival rate of 100% after 5 years. No significant differences were found in marginal bone level loss between zirconia and titanium implants. The majority of bone resorption in one-piece implants occurs within the first year of implant placement [46, 48]. Many studies have found that one-piece implants cause a wide range of marginal bone loss. The reported bone loss in these studies varied in severity between 0.28 ± 0.037 and 1.80 ± 0.28 mm [26, 41, 43, 49]. To consider an implant effective, Albrektson and Isidor [49] recommended that the mean marginal bone loss on the patient level during the first year should not exceed 1.5 mm, and 0.2 mm annually thereafter. These parameters are still regarded as the gold standard for implant success today. This prospective cohort study investigated the peri-implant marginal bone level around custom made one-piece implants. Kohal et al. [50] studied fifty-seven one-piece zirconia implants of varying length and dimeters in different intra-oral locations and reported mean marginal bone loss of 1.45 mm after 3 years (22% of the studied implants lost more 3 mm). Another study reported marginal bone loss after 1 year of 1.13 mm (14% of the implants lost more than 3 mm) [51]. Kniha et al. [52] reported 3.09 mm marginal bone loss at 1-year post-loading. However, a study reported 0.81 ± 0.77 mm as the mean marginal bone loss after 5-year recall with alumina-toughened zirconia one-piece implants [53]. They related these results to the osseoconductive surface of this implant type counteracting bone loss after healing.

To minimize variability from load or bone quality, both zirconia and titanium implants were placed in the same location for each patient. Computer software was used for accurate and reliable analysis of periapical radiographs [53]. A mean radiographic marginal bone loss of 1.31 and 1.30 mm was reported for zirconia and titanium implants respectively [54]﻿, after 12 months of functional loading. Considering 1.5 mm bone loss as the threshold for success, more than 93% of the patients were treated successfully.

One customized implant design was used in this study, further clinical studies are needed to investigate fusion sputtered zirconia implant with various micro- and macro-geometry designs such as length and diameter. Also, it is mandatory to investigate the clinical outcome of fusion sputtered zirconia implant as support for fixed dental prosthesis. Also, clinical studies are needed to study custom made zirconia implant as a root analogue. More researches are needed to study if zirconia implant used as full arch rehabilitation or not. Finally, longer term evaluation periods are required. One drawback of the present study is the placement of only one type of restoration (zirconia) which may transmit all occlusal stresses to the implants and implant-bone interface. Furthermore, non-standardized peri-apical radiographs for the assessment of the changes in the marginal bone around the implants. This has to be regarded as a limitation of the current study.

## Conclusions

Within the limitations of this clinical study, One-piece fusion-sputtered zirconia implant represents a reliable treatment modality in replacement of a missing tooth in esthetic zone, compared with one-piece titanium implants.

## Supplementary Information

**Additional file 1**. Gingival index score, Plaque index score, Periodontal depth, Marginal bone loss.

## Data Availability

The datasets used and/or analyzed during the current study available from the corresponding author on reasonable request and also supplied in the submitted Additional file 1.
