# Supplementary material for: Clinical and radiographic prospective study of customized one-piece titanium and one-piece fusion-sputtered zirconia implants: five-year mean follow-up
Source: BMC Oral Health. 2022 Nov 24;22:531. doi: 10.1186/s12903-022-02600-9 (PMC9685929; doi:10.1186/s12903-022-02600-9)
Supplement: Supplementary file 1 — Additional file 1. Gingival index score, Plaque index score, Periodontal depth, Marginal bone loss. [file 12903_2022_2600_MOESM1_ESM.docx]

**Gingival index score**

| **Material**  **Used** | **At base line** | | | | **After 6 months** | | | | **After 1 year** | | | | **After 2 years** | | | | **After 5 years** | | | |
| --- | --- | --- | --- | --- | --- | --- | --- | --- | --- | --- | --- | --- | --- | --- | --- | --- | --- | --- | --- | --- |
|  | **0** | **1** | **2** | **3** | **0** | **1** | **2** | **3** | **0** | **1** | **2** | **3** | **0** | **1** | **2** | **3** | **0** | **1** | **2** | **3** |
| **Zirconia**  **Implant** | 14 | -- | -- | -- | 13 | 1 | -- | -- | 11 | 3 | -- | -- | 11 | 3 | -- | -- | 11 | 3 | -- | -- |
| **Titanium**  **Implant** | 14 | -- | -- | -- | 13 | 1 | -- | -- | 11 | 3 | -- | -- | 11 | 2 | 1 | -- | 11 | 2 | 1 | -- |

**Plaque index score**

| **Material**  **Used** | **At base line** | | | | **After 6 months** | | | | **After 1 year** | | | | **After 2 years** | | | | **After 5 years** | | | |
| --- | --- | --- | --- | --- | --- | --- | --- | --- | --- | --- | --- | --- | --- | --- | --- | --- | --- | --- | --- | --- |
|  | **0** | **1** | **2** | **3** | **0** | **1** | **2** | **3** | **0** | **1** | **2** | **3** | **0** | **1** | **2** | **3** | **0** | **1** | **2** | **3** |
| **Zirconia**  **Implant** | 14 | -- | -- | -- | 14 | -- | -- | -- | 11 | 3 | -- | -- | 11 | 3 | -- | -- | 11 | 3 | -- | -- |
| **Titanium**  **Implant** | 14 | -- | -- | -- | 14 | -- | -- | -- | 11 | 3 | -- | -- | 10 | 3 | 1 | -- | 11 | 2 | 1 | -- |

**Periodontal depth**

| **After 5 years** | **After 2 years** | **After 12 months** | **After 6 months** | **At base line** | **No.** | **Material used** |
| --- | --- | --- | --- | --- | --- | --- |
| 3.3 | 3.2 | 3.1 | 2.7 | 2.7 | 1 | **Zirconia implant** |
| 3.3 | 3.2 | 3 | 2.8 | 2.8 | 2 |  |
| 3.5 | 3.3 | 3.1 | 2.6 | 2.6 | 3 |  |
| 3.4 | 3.4 | 3 | 2.9 | 2.9 | 4 |  |
| 3.3 | 3.3 | 3.1 | 2.6 | 2.6 | 5 |  |
| 3.5 | 3.3 | 3 | 2.8 | 2.8 | 6 |  |
| 3.1 | 3.2 | 3 | 2.7 | 2.7 | 7 |  |
| 3.3 | 3.1 | 3.1 | 2.9 | 2.9 | 8 |  |
| 3.5 | 3.5 | 3.4 | 2.6 | 2.6 | 9 |  |
| 3.2 | 3.1 | 3 | 2.5 | 2.5 | 10 |  |
| 3.5 | 3.5 | 3.3 | 2.7 | 2.7 | 11 |  |
| 3 | 3 | 2.9 | 2.6 | 2.6 | 12 |  |
| 3.2 | 3.2 | 3.1 | 2.7 | 2.7 | 13 |  |
| 3.4 | 3.4 | 3.2 | 2.9 | 2.9 | 14 |  |
| 3.3 | 3.3 | 3.2 | 2.9 | 2.9 | 1 | **Titanium implant** |
| 3.1 | 3.1 | 3.1 | 2.9 | 2.9 | 2 |  |
| 3.5 | 3.5 | 3.2 | 2.6 | 2.6 | 3 |  |
| 3.3 | 3.3 | 3.2 | 2.8 | 2.8 | 4 |  |
| 3.4 | 3.4 | 3.2 | 2.5 | 2.5 | 5 |  |
| 3.5 | 3.3 | 3.2 | 2.8 | 2.8 | 6 |  |
| 3.3 | 3.2 | 3.1 | 2.9 | 2.9 | 7 |  |
| 3.4 | 3.3 | 3.1 | 2.9 | 2.9 | 8 |  |
| 3.3 | 3.2 | 3 | 2.5 | 2.5 | 9 |  |
| 3.2 | 3.2 | 3 | 2.7 | 2.7 | 10 |  |
| 3.2 | 3.2 | 3.1 | 2.6 | 2.6 | 11 |  |
| 3.5 | 3.5 | 3.2 | 2.7 | 2.7 | 12 |  |
| 3.3 | 3.3 | 3.1 | 2.5 | 2.5 | 13 |  |
| 3.4 | 3.2 | 3 | 2.7 | 2.7 | 14 |  |

**Marginal bone loss**

| **Zirconia implant** | | | | | | | | | | | | | | |  |
| --- | --- | --- | --- | --- | --- | --- | --- | --- | --- | --- | --- | --- | --- | --- | --- |
| **After 5years** | | | **After2**  **years** | | | **After 1 year** | | | **after 6 months** | | | **At 3months** | | |  |
| D. | M. | D. | | M. | D. | | M. | D. | | M. | D. | | M. |  | |
| 1.8 | 1.7 | 1.6 | | 1.5 | 1.3 | | 1.3 | 0.7 | | 0.6 | 0.4 | | 0.3 | **1** | |
| 1.7 | 1.8 | 1.5 | | 1.6 | 1.2 | | 1.4 | 0.7 | | 0.5 | 0.2 | | 0.4 | **2** | |
| 1.7 | 1.9 | 1.6 | | 1.5 | 1.2 | | 1.3 | 0.6 | | 0.7 | 0.3 | | 0.3 | **3** | |
| 1.9 | 1.8 | 1.5 | | 1.5 | 1.2 | | 1.3 | 0.7 | | 0.6 | 0.3 | | 0.2 | **4** | |
| 1.8 | 1.7 | 1.6 | | 1.5 | 1.4 | | 1.3 | 0.6 | | 0.5 | 0.2 | | 0.3 | **5** | |
| 1.7 | 1.8 | 1.5 | | 1.6 | 1.3 | | 1.4 | 0.8 | | 0.7 | 0.3 | | 0.2 | **6** | |
| 1.8 | 1.8 | 1.5 | | 1.5 | 1.3 | | 1.3 | 0.7 | | 0.8 | 0.4 | | 0.2 | **7** | |
| 1.8 | 1.7 | 1.6 | | 1.5 | 1.3 | | 1.3 | 0.7 | | 0.6 | 0.4 | | 0.3 | **8** | |
| 1.7 | 1.8 | 1.5 | | 1.6 | 1.2 | | 1.4 | 0.7 | | 0.5 | 0.2 | | 0.4 | **9** | |
| 1.7 | 1.9 | 1.6 | | 1.5 | 1.2 | | 1.3 | 0.6 | | 0.7 | 0.3 | | 0.3 | **10** | |
| 1.9 | 1.8 | 1.5 | | 1.5 | 1.2 | | 1.3 | 0.7 | | 0.6 | 0.3 | | 0.2 | **11** | |
| 1.8 | 1.7 | 1.6 | | 1.5 | 1.4 | | 1.3 | 0.6 | | 0.5 | 0.2 | | 0.3 | **12** | |
| 1.7 | 1.8 | 1.5 | | 1.6 | 1.3 | | 1.4 | 0.8 | | 0.7 | 0.3 | | 0.2 | **13** | |
| 1.8 | 1.8 | 1.5 | | 1.5 | 1.3 | | 1.3 | 0.7 | | 0.8 | 0.4 | | 0.2 | **14** | |
| **Titanium implant** | | | | | | | | | | | | | | |  |
| **After 5years** | | | **After2**  **years** | | | **After 1 year** | | | **after 6 months** | | | **At 3months** | | |  |
| D. | M. | D. | | M. | D. | | M. | D. | | M. | D. | | M. |  | |
| 1.9 | 1.7 | 1.5 | | 1.5 | 1.2 | | 1.3 | 0.6 | | 0.7 | 0.3 | | 0.3 | **1** | |
| 1.8 | 1.8 | 1.5 | | 1.5 | 1.3 | | 1.3 | 0.7 | | 0.8 | 0.4 | | 0.2 | **2** | |
| 1.8 | 1.9 | 1.6 | | 1.5 | 1.2 | | 1.3 | 0.7 | | 0.6 | 0.3 | | 0.2 | **3** | |
| 1.8 | 1.8 | 1.6 | | 1.5 | 1.4 | | 1.3 | 0.6 | | 0.5 | 0.2 | | 0.3 | **4** | |
| 1.8 | 1.8 | 1.5 | | 1.6 | 1.3 | | 1.4 | 0.8 | | 0.7 | 0.3 | | 0.2 | **5** | |
| 1.8 | 1.8 | 1.6 | | 1.5 | 1.3 | | 1.3 | 0.7 | | 0.6 | 0.4 | | 0.3 | **6** | |
| 1.7 | 1.8 | 1.5 | | 1.6 | 1.2 | | 1.4 | 0.7 | | 0.5 | 0.2 | | 0.4 | **7** | |
| 1.9 | 1.7 | 1.5 | | 1.5 | 1.2 | | 1.3 | 0.6 | | 0.7 | 0.3 | | 0.3 | **8** | |
| 1.8 | 1.8 | 1.5 | | 1.5 | 1.3 | | 1.3 | 0.7 | | 0.8 | 0.4 | | 0.2 | **9** | |
| 1.8 | 1.9 | 1.6 | | 1.5 | 1.2 | | 1.3 | 0.7 | | 0.6 | 0.3 | | 0.2 | **10** | |
| 1.8 | 1.8 | 1.6 | | 1.5 | 1.4 | | 1.3 | 0.6 | | 0.5 | 0.2 | | 0.3 | **11** | |
| 1.8 | 1.8 | 1.5 | | 1.6 | 1.3 | | 1.4 | 0.8 | | 0.7 | 0.3 | | 0.2 | **12** | |
| 1.8 | 1.8 | 1.6 | | 1.5 | 1.3 | | 1.3 | 0.7 | | 0.6 | 0.4 | | 0.3 | **13** | |
| 1.7 | 1.8 | 1.5 | | 1.6 | 1.2 | | 1.4 | 0.7 | | 0.5 | 0.2 | | 0.4 | **14** | |
